# The Expression of CLDN6 in Hepatocellular Carcinoma Tissue and the Effects of CLDN6 on Biological Phenotypes of Hepatocellular Carcinoma Cells

**DOI:** 10.7150/jca.55727

**Published:** 2021-07-13

**Authors:** Yan Lu, Qihua Dang, Yin Bo, Xuejin Su, Liping Wang, Jiaqi Sun, Junyuan Wei, Chengshi Quan, Yanru Li

**Affiliations:** 1 The Key Laboratory of Pathobiology, Ministry of Education, College of Basic Medical Sciences, Jilin University, 126 Xinmin Avenue, Changchun, Jilin, 130021, People's Republic of China.; 2 The Department of Anatomy, College of Basic Medical Sciences, Jilin University, 126 Xinmin Avenue, Changchun, Jilin, 130021, People's Republic of China.; 3 The Department of Pathology, Jilin Provincial Cancer Hospital, 1018 Huguang Road, Changchun, Jilin, 130021, People's Republic of China.

**Keywords:** CLDN6, human hepatocellular carcinoma, proliferation, migration, invasion, EMT

## Abstract

CLDN6, a member of claudin (CLDN) family, was found to be a breast cancer suppressor gene in our early experiments. However, CLDN6 was highly expressed in human hepatocellular carcinoma (hHCC) (TCGA database), and the role of CLDN6 in hHCC is still unclear. To investigate the expression of CLDN6, immunohistochemical staining was performed in hHCC tissues. As a result, hHCC tissues highly expressed CLDN6, and the expression was related to the degree of tumor's differentiation. To research the role of CLDN6 in hHCC cells, CLDN6 was silenced in HepG2 and Hep3B cells which highly expressed CLDN6 through liposome transfection. Results showed that after silencing of CLDN6, the proliferation, migration and invasion abilities of hHCC cells were inhibited. Meanwhile, the expression of E-cadherin was upregulated, and the expression of N-cadherin and Vimentin was downregulated. All the results above indicated that CLDN6 promoted the development of hHCC, and could be a potential target for the treatment of it.

## Introduction

Tight junctions (TJs) locate in the most apical part of the junctional complexes between neighboring epithelial and endothelial cells 1, and contribute to the maintenance of cell polarity and barrier function 2, 3. TJs mainly consist of two types of proteins, one of which is structural membrane proteins, including occludin, claudin (CLDN), and junctional adhesion molecules (JAMs) 4. CLDNs are thought to be the key structural integral components of tight junctions 5. In a number of cancers, the structure and function of tight junctions are abnormal 6. A large number of studies showed that CLDNs present as up-regulated expression or down-regulated in malignant tumor derived from epithelium. The direct effect of this abnormal expression is to destroy the composition ratio of closely connected components and cause changes in the structure and function of closely connected components. For example, the destruction of tight junction integrity leads to the increase of cell gap, the disappearance of cell polarity and the increase of invasiveness. This abnormal expression can also indirectly regulate the proliferation, differentiation and apoptosis of tumor cells through signal transduction, thus playing an important role in tumor occurrence, invasion and metastasis. Abnormal expression of CLDNs has been considered to be one of the mechanisms of the malignant progression of cancer 7. Moreover, it is believed that this differential expression of CLDNs is related to its highly tissue-specific 8, 9.CLDNs can be used as a marker for tumor diagnosis and an independent indicator for prognosis observation and tumor recurrence 10-12. For example, the expression of CLDN1 is low and expression of CLDN2 is high in endometrioid hyperplasia tissues. On the contrary, in plasma papillary adenocarcinoma, CLDN1 is highly expressed and CLDN2 is low 13. In ovarian cancer tissues, four markers including CLDN3 can be used as accurate diagnostic indicators 14. However, the tissue specific mechanism of CLDNs expression changes in tumors is still unclear, which may be related to precise molecular regulation.

CLDN6, one of 28 members of the CLDNs family 15, was first found and confirmed as a breast cancer suppressor gene. Overexpression of CLDN6 can inhibit the proliferation, migration and invasion of breast cancer cells 16. Similarly, overexpression of CLDN6 can inhibit the invasive phenotype of cervical cancer cell lines 17.

Primary liver cancer refers to the malignant tumor occurring in intrahepatic bile duct epithelial cells or liver cells 18, hepatocellular carcinoma (HCC) accounts for 90% of the cases. It has been reported that CLDN6 can increase the ability of hepatitis C virus (HCV) to enter liver cells, thereby increasing the degeneration and necrosis of liver cells 19, 20. In previous experiments, we found that CLDN6 was highly expressed in human liver cancer tissues, but the expression of CLDN6 in human liver cancer cells and its specific role in the biological behavior of liver cancer cells are still unclear. Therefore, this study will explore the clinical significance in liver cancer tissues of CLDN6 and its effects on the proliferation, migration and invasion abilities of human hepatocellular carcinoma (hHCC) cell lines HepG2 and Hep3B.

## Materials and Methods

### Cell culture

Two hHCC cell lines (HepG2 and Hep3B) and human normal liver cell line (LO2) were cultured in high glucose DMEM medium (Gibco, Carlsbad, California, USA) supplemented with 10% fetal bovine serum (FBS; Hyclone, Logan, UT, USA) at 37 ºC and in the presence of 5% CO_2_.

### Patient tissue samples

48 human liver cancer samples including 13 specimens with highly differentiated, 20 specimens with moderately differentiated, 15 specimens with poorly differentiated were collected from the pathology department of Jilin provincial cancer hospital with approval by the institutional review board (IRB). None of the patients received neo-adjuvant therapy. The patients' medical records were reviewed to obtain their age, tumor status and clinical stage. All tissues were micro-dissected and confirmed by sequential pathological analysis of paraffin sections. The stage and grade of the tumors were evaluated according to the International Federation of Gynecology and Obstetrics (FIGO) staging system. All the clinical materials have been obtained with prior consent and approval from patients.

### Immunohistochemistry

Paraffin sections of human liver cancer were dewaxed using xylene and gradient alcohol. Then, the sections were immersed in citrate buffer (pH = 6.0) (95°C - 98°C, 5 min) for antigen retrieval. The sections were treated with 3% hydrogen peroxide (H_2_O_2_) for 30 min to inactivate endogenous peroxidase activity. Sections were blocked with normal goat serum for 1h and then incubated with rabbit anti-human CLDN6 (1:200 dilution, Abcam, USA) at 4°C overnight. Sections were then washed with PBS and incubated with biotinylated goat anti-rabbit secondary antibody at 37°C for 30 min. Streptomycin anti-biotin-peroxidase was added and the sections were incubated at room temperature for 20 min. Diaminobenzidine (DAB) was used for color development. Sections were counterstained with Mayer's haematoxylin. The negative controls were handled in the same way except that the cells were incubated in PBS instead of primary antibody.

CLDN6 was expressed in cell membrane/cytoplasm, and yellow or brown-yellow staining in cell membrane/cytoplasm was positive. The staining was graded for intensity (0-negative, 1-weak, 2-moderate, and 3-strong) and percentage of positive cells as follows: 0, <5% positive tumor cells; 1, 5%-25% positive tumor cells; 2, 26%-50% positive tumor cells; 3, >50% positive tumor cells. The cell counting was repeated in five randomly-selected microscopic fields at ×200 magnification. The total score of each section: 0-3, negative; 4-6, positive.

### Reverse Transcription-Polymerase Chain Reaction (RT-PCR)

Total RNA was extracted using TRIzol reagent (Invitrogen, USA). 0.5 μg RNA was reverse-transcribed with PCR diagnosis kits (TianGen, China) and random 9-mer primers (TAKARA, Japan). PCR reaction system contains 50 ng cDNA, 1 μl of each primer and 1×PCR master Mix (TAKARA). PCR cycling conditions were as follows 94 ºC for 5 min, followed by 35 cycles at 94 ºC for 30 s, 56 ºC for 30 s, and 72 ºC for 30 s with a final extension step of 10 min at 72 ºC. The expression levels of β-actin mRNA were measured as the internal control. The sequences of primers were as follows: CLDN6: forward, 5'-TTCATCGGCAACAGCATCGT-3' and reverse, 5'-GGTTATAGAAGTCCCGGATGA-3' (amplification length, 345 bp); β-actin, forward, 5'-TACCTCCCAAGTCCTGTATGAG-3' and reverse, 5'-TGAGCAGCATCAAACTGTGTAG-3' (amplification length, 180 bp). Reactions were carried out in triplicate. The results were analyzed using Quantity One 4.4.1 software (Bio-Rad Laboratories Inc, Hercules, California, USA).

### Western blot analysis

Total protein was extracted using 400 μl ice-cold Radio Immunoprecipitation Assay Lysis Buffer (HuaTe Sheng, China) with 40 μl (10 mM) phenylmethanesulfonyl fluoride (PMSF) according to the supplier's instructions. The concentration of the total protein was determined using BCA Protein Assay Kit (Beyotime Biotechnology, Shanghai, China). 50 μg of total proteins of each sample per lane were resolved by 12% SDS-PAGE and then transferred onto PVDF membrane (Millipore, USA). Membranes were blocked and probed with primary antibodies: CLDN6 (1:1000 dilution Bioworld, USA), β-actin (1:2000 dilution, Santa Cruz Biotechnologies, California, USA). Membranes were then washed and incubated with HRP-conjugated secondary antibodies (1:2000 dilution, Protein Tech, USA). Immunoreactive bands were detected using ECL western blot reagents (GE, Fairfield, Connecticut, USA). Experiments were repeated independently three times.

### Immunocytochemistry

When the cells reach 80% confluence on glass cover slips and were fixed with preheated 4% paraformaldehyde for 25 min at room temperature. The coverslips were immersed in citrate buffer (pH = 6.0) (95-98ºC, 5 min) and then incubated in 3% H_2_O_2_ (room temperature, 15 min) for antigen retrieval. Cells were blocked with 1% BSA (serum albumin, Beyotime Biotechnology, Shanghai, China) at room temperature for 20 min and then incubated with rabbit anti-human CLDN6 antibody (1:200 dilution, Abcam, USA) overnight at 4°C. The cells were washed with PBS and incubated for 30 min with biotinylated goat anti-rabbit secondary antibody at 37°C. Streptomycin anti-biotin-peroxidase solution was added to each coverslip and incubated for 30 min at room temperature. Diaminobenzidine (DAB) was used for color development. Cells were counterstained with Mayer's haematoxylin. The negative controls were handled in the same way except that the cells were incubated with PBS instead of first antibody.

### Immunofluorescence staining

When the cells reached 80% confluence, 4% paraformaldehyde was used to fix the cells for 15 min at room temperature and the cells were washed three times. Primary anti-CLDN6 antibody was applied at 1:200 dilution to cells at 4ºC overnight. Phycoerythrin (PE)-conjugated anti-rabbit IgG secondary antibody (1:200 dilution, Cell signal technology, USA) was then applied for 30 min at room temperature. After washing by PBS, nuclei were counterstained with DAPI (5 μg/ml, Sigma, USA) for 5 min in the dark. Negative control sections were immunostained as described above except that the cells were incubated with PBS instead of first antibody. Cells were visualized with a fluorescence microscope microscopy (Olympus, Tokyo, Japan).

### Plasmid construction and transfection

Short hairpin RNA (shRNA) oligonucleotides targeting CLDN6 (NM_021195) and negative control were individually cloned into pGCsilencer™U6/Neo/GFP vectors (GeneChem, Shanghai, China). Target sequence: ca GTGCAAGGTGTACGACTCA TTCAAGAGA TGAGTCGTACACCTTGCACtg.

HepG2 and Hep3B cells were transfected with shRNA-CLDN6 using SuperFect Transfection Reagent (QIAGEN, USA). After transfection for 48 h, medium containing 500 g/ml G418 (Sigma, St. Louis, Missouri, USA) was used for screening. After 1-2 weeks of G418 screening, cells were infinitely diluted to the 96-well plate. In other words, there was only one cell in each well of the plate. The monoclonal cells were cultured and expanded.

### Flow cytometry cell cycle analysis

When fused to 80%, cells were collected and fixed with 70% precooled ethanol for 24h at -20°C. Cells were washed twice with precooled PBS, treated with RNase A (37°C, 30 min) (Fermentas) and propidium iodide (PI, Sigma) (4°C, 30 min). Cell cycle distribution was observed at 488 nm of excitation wavelength by flow cytometry (Becton & Dickinson, USA) and photographed.

### CCK8 assay

Cells were seeded in 96-well plates at a low density (1, 000 cells /well) and cultured as described above. Every 24h over the next 6 days, Cell Counting Kit-8 (CCK8, Dojindo, Tokyo, Japan) solution (10%) was added into each well, and incubated at 37°C. After 2 h, the optical density (OD) of each well at 450 nm wavelength was recorded on a Microplate Reader (Thermo, Varioskan Flash). Five replicate counts were performed in each of three independent experiments.

### Wound-healing assay

5×10^5^ cells were seeded per well into a 6-well plate and cultured as described above. When cells had grown to confluence, a straight wound was created in each monolayer by dragging a 200 μl pipette tip through the monolayer. Cells were washed with PBS and then cultured in serum-free medium. Images were taken under a microscope (Olympus, Japan) at 0h and 24h after wounding to determine the width of the wounded area. The relative migration distance (% of recovery) was calculated as (W_0_-W_24_)/ W_0_×100%. The migrated cells in each well were counted in five different fields per experiment under the microscope.

### Migration and Invasion assays

Migration assay was performed using Transwell chamber (Corning, Lowell, MA, USA) without coating with Matrigel. Cells were collected and adjusted to 1×10^5^/ml. 200 μl cell suspension was added into each upper chamber, and 500 μl medium (containing 20% FBS) was placed into the lower compartment as a chemoattractant. Cells were incubated at 37 ºC and in the presence of 5% CO_2_ for 24 h, and then stained with 1% crystal violet and counted.

Invasion assays were utilized using Transwell chamber (Corning) coating with Matrigel (BD Biosciences, San Jose, USA). 1×10^5^/200 μl cell suspension was added into each upper chamber, and 500 μl medium (containing 20% FBS) was placed in the lower compartment as a chemoattractant. After 24 h, the invasive cells were stained and counted.

### Statistical analyses

All data were expressed as mean ± standard deviation (SD). Student's t-test was employed with SPSS version 18.0 software to determine the significance of differences between two groups, and *p*<0.05 was considered significant.

## Results

### CLDN6 is highly expressed in hHCC

**Figure 1:**
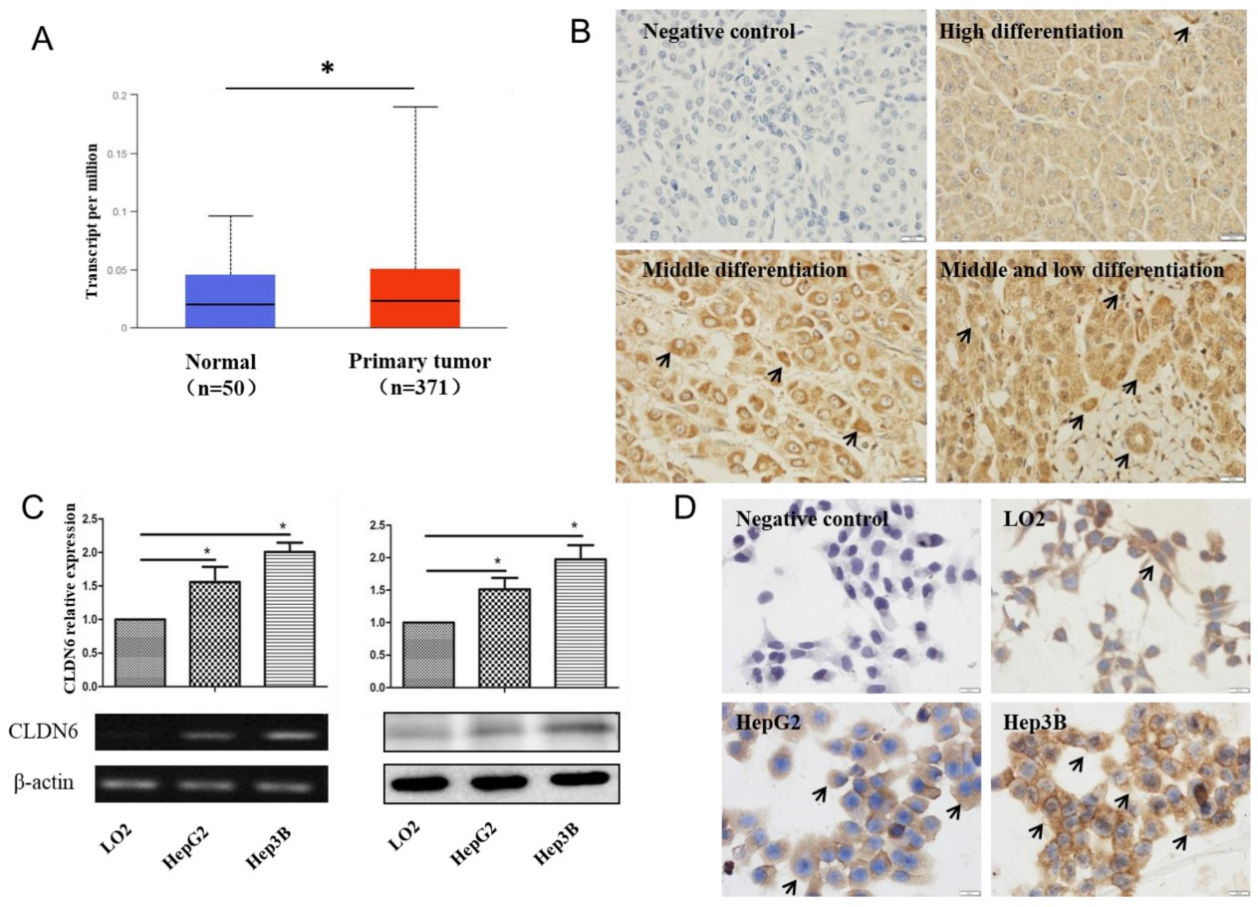
** The expression of CLDN6 in hHCC tissues and cells.** A, TCGA database was used to analyze CLDN6 expression in hHCC patients. B, immunohistochemical staining technology was used to determine the expression of CLDN6 in hHCC tissues. C, RT-PCR and western blotting analysis was performed to determine the expression of CLDN6 in hHCC cells HepG2 and Hep3B. D, immunocytochemistry was utilized to detect the expression and location of CLDN6 in hHCC cells HepG2 and Hep3B, the black arrows point out typical cells that express CLDN6. (*p < 0.05 is considered statistically significant). Bars represent mean ± SE (n = 3).

In order to analyze the expression of CLDN6 in hHCC, we used the data of hHCC patients in the TCGA database for analysis, which contains 52 normal tissues and 503 HCC tissues. The results showed that CLDN6 is highly expressed in hHCC tissues compared with normal tissues (Fig. 1A). Furthermore, we detected the expression of CLDN6 in 48 hHCC tissues using immunohistochemistry. The results showed that CLDN6 was highly expressed in hHCC and mainly expressed in the cell membrane and the cytoplasm (Fig. 1B). Positive expression of CLDN6 protein was found in 79.17% (38/48) of hHCC tissues. The relationship between CLDN6 and clinicopathological parameters was evaluated and the results were summarized in Table 1. The positive expression of CLDN6 in hHCC tissues was related to the degree of tumor differentiation. No significant association was found between CLDN6 and tumor stage, lymph node metastasis, gender or age.

Semi-quantitative RT-PCR and western blot were utilized to detect the expression of CLDN6 in human normal liver cells and hHCC cells. As a result, the mRNA and protein level of CLDN6 was higher in HepG2 and Hep3B as compared with normal liver cells LO2 (Fig. 1C). Immunocytochemical staining assay showed that CLDN6 was strongly positively expressed in HepG2 and Hep3B and mainly expressed on the cell membrane and cytoplasm (Fig. 1D).

### Establishment of hHCC cell line with silencing of CLDN6

**Figure 2:**
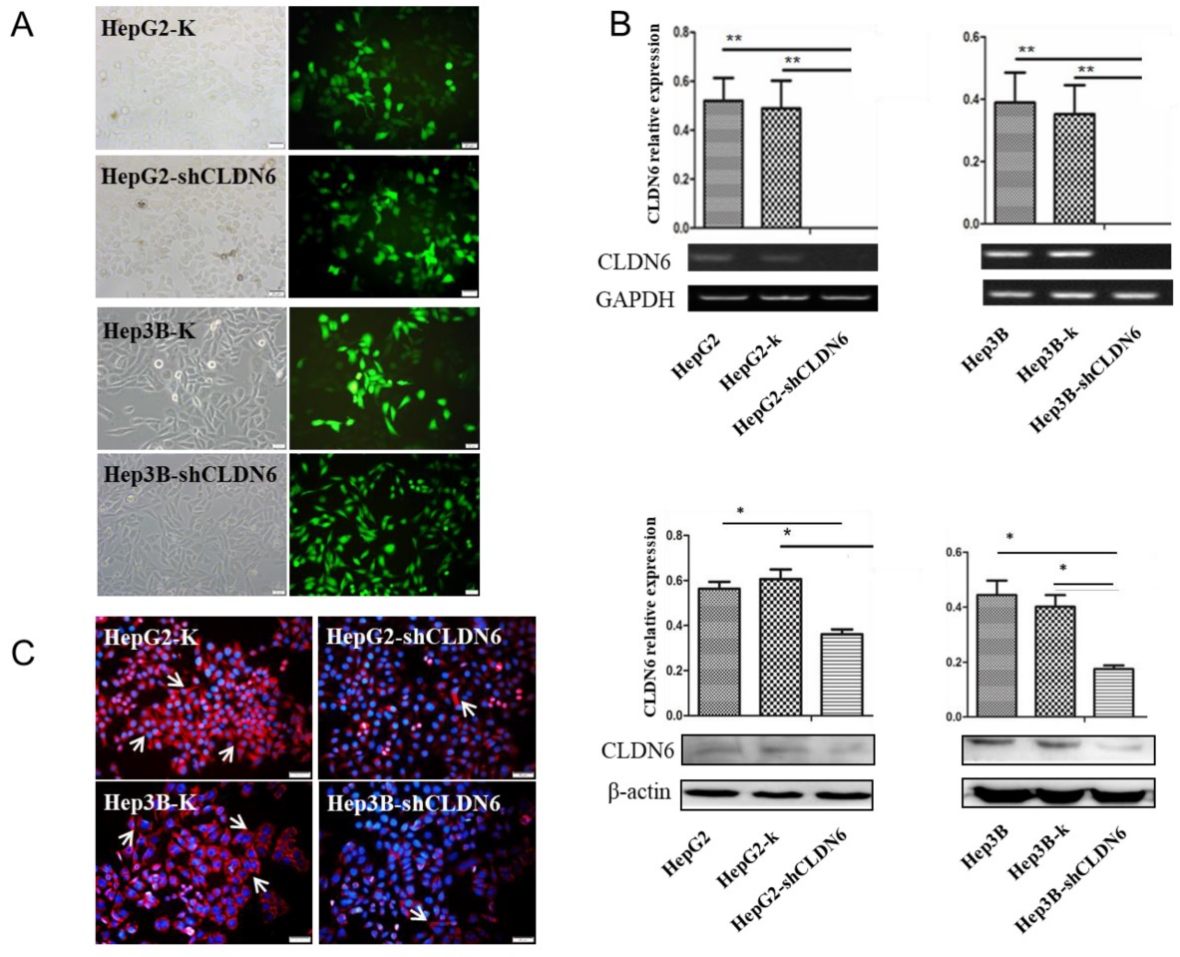
** The silencing of CLDN6 in hHCC cells HepG2 and Hep3B.** A, GFP efficiency of HepG2 and Hep3B cells after silencing of CLDN6. B, RT-PCR and western blot results for the expression of CLDN6 in HepG2 and Hep3B cells. C, Immunofluorescence of CLDN6 expression and location in HepG2 and Hep3B cells, the white arrows point out typical cells that express CLDN6. (bar, 20 μm; *, *p* < 0.05; **, *p* < 0.01).

CLDN6 is highly expressed in hHCC cells, indicating that CLDN6 is probably related to the biological behavior of the cells. To research the role of CLDN6 in hHCC cells, we used liposome transfection to silence CLDN6 gene, and obtained stable clones by G418 screening. Green fluorescence was highly expressed in the transfected cells (Fig. 2A), suggesting a high transfection efficiency. The expression of CLDN6 was further confirmed by semi-quantitative RT-PCR, western blot (Fig.2B), and Immunofluorescence staining (Fig. 2C). The results showed that the mRNA and protein level of CLDN6 in HepG2-shCLDN6 and Hep3B-shCLDN6 were significantly lower than that in control cells. Immunofluorescence staining showed that CLDN6 was located in cell membrane and cytoplasm of the cells.

### Silencing CLDN6 inhibits the proliferation, migration and invasion capacity of hHCC cells

**Figure 3:**
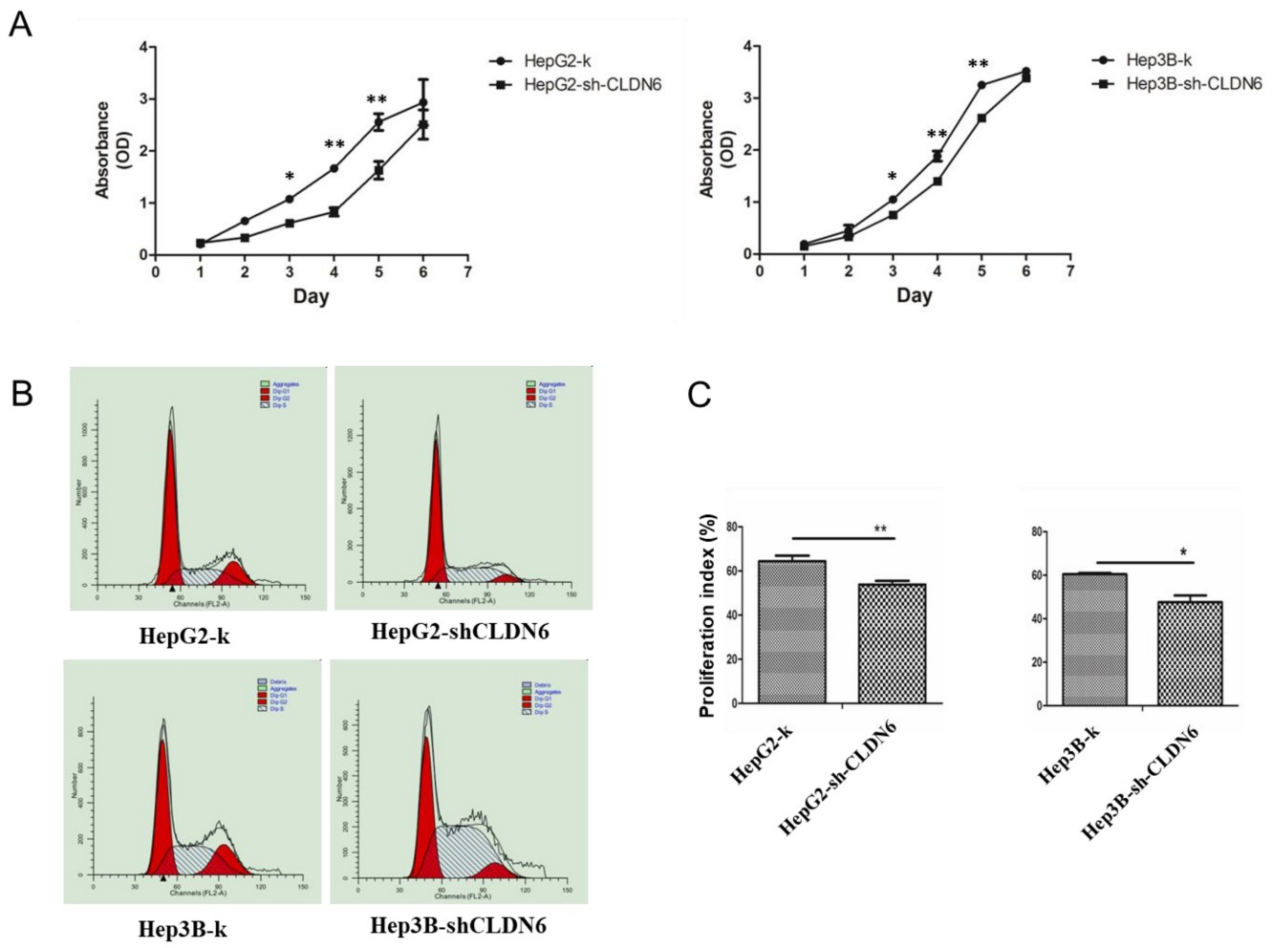
** Silencing of CLDN6 decreased proliferation capacity in hHCC cells HepG2 and Hep3B.** A, CCK-8 assay for HepG2 and Hep3B cells after CLDN6 silencing. B, effects of CLDN6 on HepG2 and Hep3B cell cycle phase distribution. C, PI ((S+G2) / (G1+S+G2) × 100%) of the HepG2 and Hep3B cells after CLDN6 silencing. “dip” is the abbreviation for diploid. (*, *p* < 0.05; **, *p* < 0.01).

To examine the effect of CLDN6 on the proliferation capacity of HepG2 and Hep3B cells, CCK-8 assay was performed. As a result, the cell growth rate over a 6-day period was significantly decreased in HepG2-shCLDN6 and Hep3B-shCLDN6 cells compared with that of control (Fig. 3A). To further clarify the influence of CLDN6 on the cell cycle and proliferation, the proliferative index (PI) of HepG2-shCLDN6 and Hep3B-shCLDN6 cells were compared with those of control. As shown in Fig. 3B, HepG2-shCLDN6 and Hep3B-shCLDN6 cells displayed an extended G1 phase and a shortened G2 and S phases (Fig. 3B), and PI was lower in HepG2-shCLDN6 and Hep3B-shCLDN6 cells (Fig. 3C).

**Figure 4:**
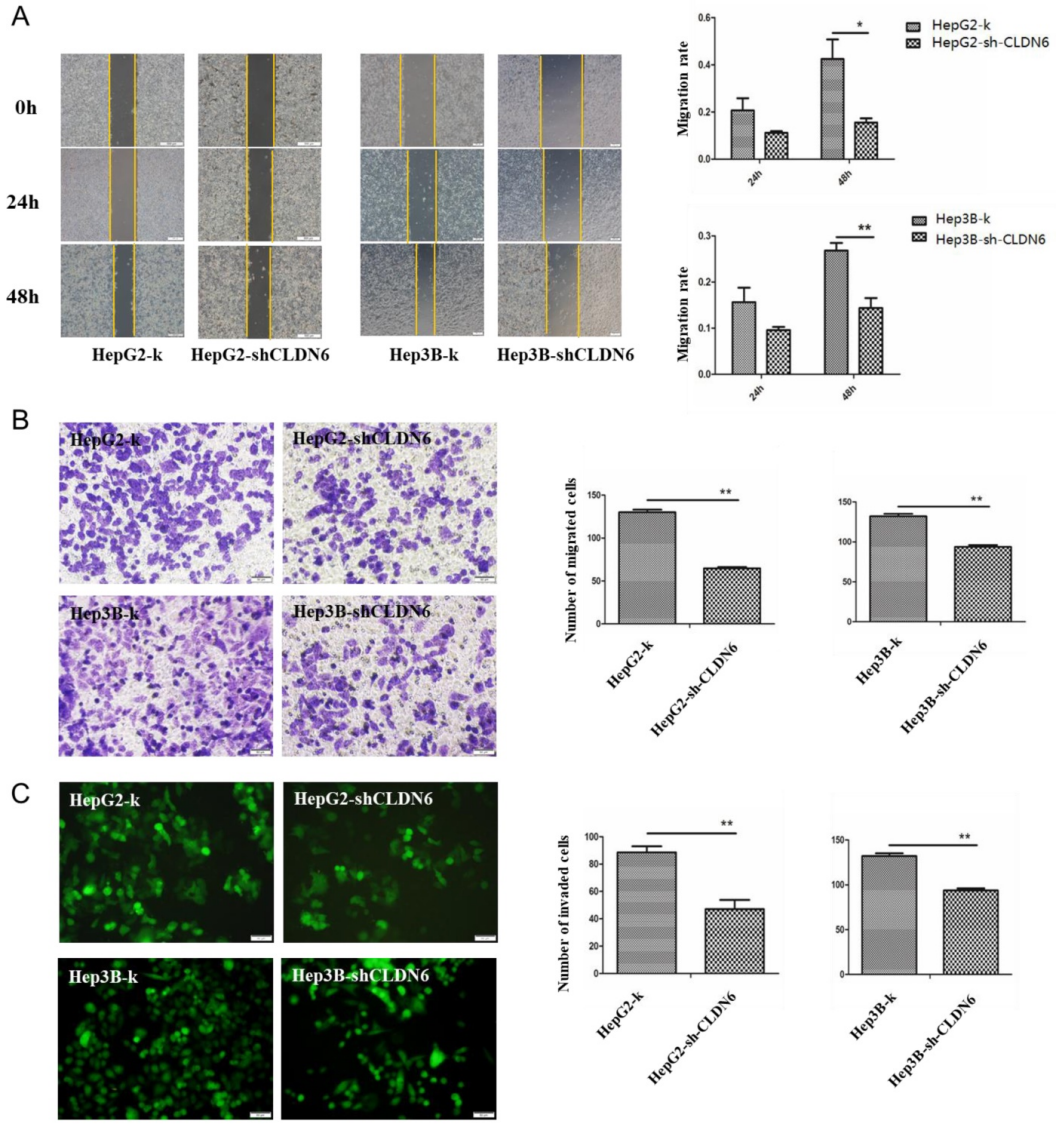
** Silencing of CLDN6 decreased the migration and invasion capacity in hHCC cells HepG2 and Hep3B.** A, representative light microscope images of wound-healing assays for HepG2 and Hep3B cells after CLDN6 silencing to evaluate their migration rate into the cell-free area (bar, 200 μm).B, Transwell assay (without Matrigel), cells that invaded through the Matrigel were stained with Giemsa and counted (bar, 50 μm) C, Matrigel invasion assay (bar, 50 μm). All results are presented as the average of cells counted in 10 fields per condition. (*, *p* < 0.05; **, *p* < 0.01).

It is known that CLDN6 participates in cellular TJ formation and TJ stability 21. Thus, downregulation of CLDN6 could lead to a different invasive phenotype in cancer cells. To detect the effect of CLDN6 on the migration ability of HepG2 and Hep3B cells, wound-healing and Transwell without Matrigel chamber assay was utilized. Wound-healing assay showed that compared with the control group, the migration ability of HepG2-shCLDN6 and Hep3B-shCLDN6 cells was significantly decreased, which was most markedly at 48h (Fig.4A). Transwell chamber without Matrigel results showed that the number of migrated cells in HepG2-shCLDN6 and Hep3B-shCLDN6 was significantly decreased after 24h compared with that of control (Fig. 4B).

To detect the effect of CLDN6 on the invasion ability of HepG2 and Hep3B cells, Transwell assay with Matrigel was performed. The results showed that the number of HepG2-shCLDN6 and Hep3B-shCLDN6 cells crossing the membrane was significantly decreased after 24h compared with the control group (Fig. 4C).

These observations showed that the proliferation, migration and invasion abilities of hHCC cells were decreased after CLDN6 silence, suggesting that CLDN6 promotes malignant phenotype of hHCC cells.

### Silencing CLDN6 inhibited the epithelial-mesenchymal transition (EMT) of hHCC cells

**Figure 5:**
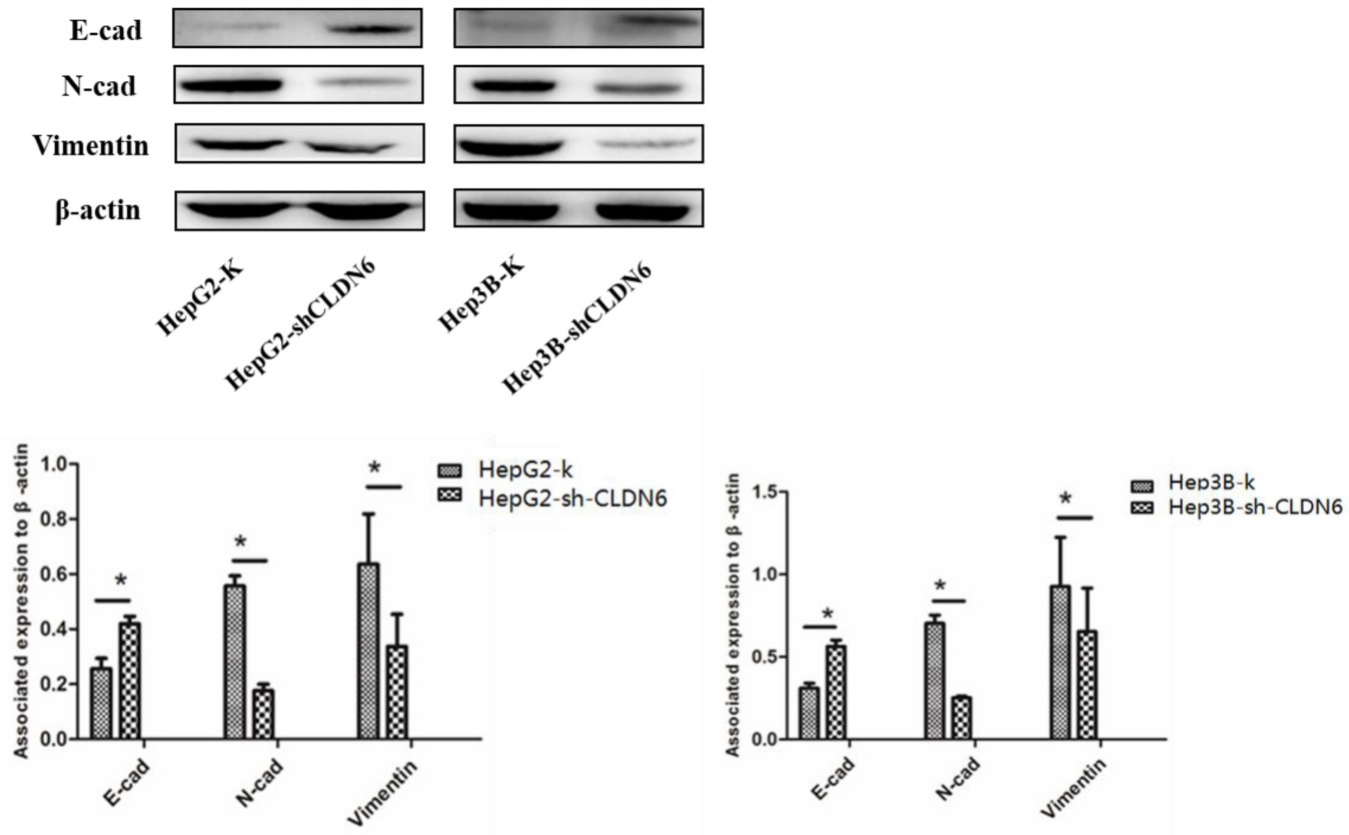
** Silencing of CLDN6 inhibited EMT in HepG2 and Hep3B cells.** Western blotting analysis was used to determine the expression of EMT-related proteins (E-cadherin, N-cadherin, and vimentin) in HepG2 and Hep3B cells. Results of densitometry analysis of relative expression levels after normalization to loading control β-actin are presented. (*, *p* < 0.05).

EMT plays an important role in tumor metastasis 22, and is one of the mechanisms of tumor migration and invasion 23. The down-regulation of epithelial genes and up-regulation of mesenchymal genes in carcinoma cells promote an invasive and metastatic phenotype 24. To investigate the role of CLDN6 on EMT, we examined the changes in EMT associated proteins E-cadherin, N-cadherin and Vimentin in HepG2 and Hep3B cells after silencing CLDN6. The results showed that the expression of E-cadherin was upregulated, while the expression of N-cadherin and Vimentin was downregulated in HepG2-shCLDN6 and Hep3B-shCLDN6 cells (Fig. 5). Therefore, silencing CLDN6 suppressed EMT, and subsequently inhibited the migration and invasion of breast cancer cells.

## Discussion

CLDN6, as a member of the CLDNs family, has a significant influence on malignant phenotypes of tumors due to its abnormal expression 25. This study mainly investigated the clinical significance of CLDN6 expression in human liver cancer tissues and the effect of CLDN6 on the tumor biological behavior of hHCC cells HepG2 and Hep3B through EMT.

CLDN6 expression has obvious tissue specificity. Through TCGA database, we found that CLDN6 is highly expressed in hHCC tissues compared with normal tissues, indicating that the expression of CLDN6 in hHCC tissues was upregulated, which was consistent with previous report with 10 hHCC tissue samples and matched tumor-adjacent tissue samples 26. In order to further explore the relationship between the expression of CLDN6 and the development of hHCC, we detected the expression of CLDN6 in 48 hHCC tissues by immunohistochemical staining technology. The results showed that the positive expression rate of CLDN6 was 79.17%. Meanwhile, through analysis, we found for the first time that the high expression of CLDN6 in hHCC tissues was related to the degree of tumor differentiation, but not related to tumor stage, lymph node metastasis, gender or age of patients. Therefore, we speculated that the upregulated expression of CLDN6 in liver cancer tissues may promote the occurrence and development of hHCC. The expression of CLDNs in epithelial cells is a dynamic equilibrium pattern, high expression of some CLDN member proteins leads to a low expression of other members of the CLDN family 27. The up-regulation of CLDN6 destroyed the dynamic equilibrium pattern of TJs, and subsequently damaged the structure and functions of TJs, and subsequently damaged the structure and function of TJs. The disorder of TJs leads to the loss of intercellular adhesion, decreases cohesion of cancer cells, causes poor differentiation and increases invasiveness, thus promoting the occurrence and metastasis of tumors, leading to loss of cell polarity and abnormal inflow of growth factors, and stimulating the carcinogenesis of epithelial cells through autocrine and paracrine.

The mRNA and protein expression levels of CLDN6 were detected by RT-PCR, western blot and immunocytochemistry. The results showed that CLDN6 was highly expressed in hHCC cell lines HepG2 and Hep3B, and was mainly expressed in the cell membrane and cytoplasm. Similarly, CLDN6 was highly expressed in endometrial carcinoma cell line HEC-1-B 28. This is different from the low expression of CLDN6 in other digestive system tumors, such as gastric 29 and colon cancer 30. This can be attributed to tissue and cell specificity of expression and distribution of CLDNs, which is the main reason for different TJ functions in different types of epithelial tissues 31. Similarly, the distribution of CLDNs differs greatly in different structures within the same organization 32, 33.

In order to further clarify the role of CLDN6 in hHCC, we silenced the CLDN6 gene in HepG2 and Hep3B cells by liposome transfection. Compared with the control group, the proliferation, migration and invasion abilities of HepG2 and Hep3B cells were significantly reduced after CLDN6 silencing. These results indicated that CLDN6 promoted the proliferation, migration and invasion of hHCC cells. This is consistent with some conclusions reported in the previously that the high expression of CLDNs in tumors is related to the high invasion and metastasis abilities of tumors. For example, CLDN2 is highly expressed in liver metastasis tissues of breast cancer and enhances adhesion to extracellular matrix through type IV collagen and fibronectin, thereby promoting liver metastasis of breast cancer cells 34.

EMT refers to the biological process in which epithelial cells are transformed to mesenchymal phenotypic cells through specific procedures and plays an important role in tumor metastasis 35. During EMT, epithelial cells lose cell polarity, connection with basement membrane and other epithelial phenotypes, and obtain mesenchymal phenotypes with high migration and invasion abilities 36, 37. Previously, we found that CLDN6 suppress EMT in breast cancer cells 38. In order to investigate what effect of CLDN6 has on EMT in hHCC cells, we utilized western blot to detect the changes of EMT-related protein expression in HepG2-shCLDN6 and Hep3B-shCLDN6 cells. The results showed that the expression of E-cadherin was upregulated and the expressions of N-cadherin and vimentin were downregulated in HepG2-shCLDN6 and Hep3B-shCLDN6 cells, suggesting that CLDN6 can promote EMT in hHCC. So CLDN6 may regulate EMT through different signal pathway in different tumors. Consistent with our data, CLDN1 suppressed E-cadherin and subsequently induced EMT in hepatocellular carcinoma cells 39. However, CLDN3, CLDN4 40, and CLDN7 41 have been shown to suppress EMT in ovarian carcinoma cells and lung cancer cells.

In conclusion, CLDN6 plays an active role in promoting the development of hHCC, and has the potential to be a biomarker of hHCC metastasis.

## Figures and Tables

**Table 1:** Relationship between CLDN6 expression and clinicopathological parameters

Item	n	CLDN6 (+)	CLDN6 (-)	*p*
**Differentiated degree**				
Highly differentiated	13	6	7	0.03^*^
Moderately differentiated	20	18	2	
Poorly differentiated	15	14	1	
**Clinical stages**				
I-II	39	30	9	0.661
II-IV	9	8	1	
**Lymphatic metastasis**				
+	3	2	1	0.203
-	45	36	9	
**Age (year)**				
≤55	22	18	4	0.735
>55	26	20	6	
**Gender**				
Man	36	27	9	0.414
Woman	12	11	1	

^*^p<0.05
